# The Impacts of Breakthrough Drug Classes on Total Healthcare Expenditures

**Published:** 2016-02-26

**Authors:** Zeynal Karaca, Steven N. Wiggins

**Affiliations:** 1 Agency for Healthcare Research and Quality (AHRQ), Center for Delivery, Organization and Markets, Rockville, MD, USA; 2 Texas A & M University, Economics Department, College Station, TX, USA

**Keywords:** meps, prescription drugs, healthcare expenditures, drug classes

## Abstract

**Background:** Pharmaceutical firms spend billions of dollars developing new therapies, which are often sold at a substantial premium over older therapies. The costs and benefits of these newer and more expensive drugs, however, are controversial. The empirical evidence on whether newer drugs can decrease overall healthcare expenditures may enable private and public health policymakers to develop new drug benefit structures.

**Objective:** This paper investigates the impact of new drugs and drug classes on overall healthcare expenditures. The paper focuses on the level of reductions in total healthcare expenditures drawn from the replacement of other drugs with breakthrough drug classes.

**Methods:** We used the Medical Expenditure Panel Survey (MEPS) data sets from 1996 to 2001. To measure the effect of important drugs prescribed for a given condition on the expenditures, we developed a model that captures the effect of important drugs on expenditures in a less restrictive way than the extant literature. Our analysis of these drug groupings offers several improvements over prior work. First, we treated all drugs with a similar pharmacology as the same, rather than assigning their therapeutic value based on year of introduction. Second, our approach recognizes that innovations emerge in waves and that drugs within a particular group are more similar therapeutically to each other than to other existing drugs, or other drugs introduced in the same or different years. Third, we separately estimated the effects of each of these groups of drugs on drug and non-drug expenditures. Finally, we measured the cost impact of drug use in a more general way than in the existing literature. We examined six different groups of breakthrough drugs, selective serotonin reuptake inhibitors, statins, angiotensin-converting-enzyme (ACE) inhibitors, histamine type 2 antagonists, proton pump inhibitors, calcium channel blockers and fluoroquinolones.

**Results:** Our results demonstrate that drugs from breakthrough classes - except ACE inhibitors - are more expensive than other drugs. Next, we measured the impact of breakthrough drug classes and other drugs on total non-drug medical expenditures. The results indicate that important drugs significantly decrease total non-drug expenditures for all breakthrough classes, except fluoroquinolones. In general, the reduction in non-drug expenditures is many times larger than the increased drug expenditures.

**Conclusion:** Breakthrough drug classes, with the exception of fluoroquinolones, substantially reduce overall healthcare expenditures.

## 1. Background

The impact of new drugs and drug classes on overall healthcare expenditures is an important medical and economic question. Pharmaceutical firms spend billions of dollars developing new therapies, which are often sold at a substantial premium over older therapies. The use of these newer and more expensive drugs, however, is controversial. Health plans, Medicare and Medicaid often restrict the use of newer therapies due to cost considerations. These policies may reduce pharmaceutical costs, but run the risk of excluding valuable therapies that can lower overall healthcare spending, reduce morbidity, lost schooling and work, and hospital stays. Others argue that newer therapies cost more and there are few differences between their therapeutic effects as compared to older, cheaper therapies.

In an important paper, Lichtenberg[1] shows that people taking newer drugs pay more, but experience even larger non-drug cost savings, and experience fewer lost work-days than people using older drugs. His results indicate that newer drugs reduce overall medical costs and improve health. Lichtenberg[2] updates the original study by incorporating new observations from the 1997 and 1998 Medical Expenditure Panel Survey (MEPS) datasets, and by analyzing the sample for the entire population and for just the Medicare population. The new results parallel his previous findings, but the effect of drug age on medical expenditures is found to be even higher, showing that newer drugs decrease non-drug expenditures by about seven times more than the increase in drug expenditures. Lichtenberg[3] also found that the mean age of drugs used by Medicare enrollees with private prescription insurance is about 9% lower than the mean age of drugs used by Medicare enrollees without private or public prescription insurance.

Duggan[4] has criticized the argument that the "replacement of older drugs by newer drugs may lower healthcare spending by reducing the demand for hospitalizations and other healthcare services." Duggan investigates antipsychotic drugs and shows that newer antipsychotic drugs increase prescription drug expenditures by 610%, but do not reduce spending on other types of medical care services.

Similarly, Zhang and Soumerai[5] has criticized Lichtenberg's findings by claiming that the original findings of Lichtenberg[1,2] were not maintained under plausible alternative assumptions. The authors suggest a more rigorous research on specific drugs and conditions to assess the impact of newer drugs on total healthcare costs.

These results call into question certain of Lichtenberg's conclusions. Most important, Duggan shows that, at least for antipsychotics, the use of new drugs does not appear to be linked to reduced healthcare expenditures. This conclusion raises questions about Lichtenberg's methodology. In particular, Lichtenberg assumes that all newer drugs of a similar age represent comparable improvement over older drugs of a similar age. That is, drug improvement is linear in time and the rate of improvement is the same across all classes. This assumption is strong and potentially could lead to biased estimation of the impact of drug age on drug and non-drug expenditures. More generally, the Duggan[4] results raise the question of whether newer drugs generally reduce non-drug expenditures, as found by Lichtenberg,[1,2] or whether such reductions occur in only certain therapeutic classes. Zhang and Soumerai[5] noted similar limitations, but they supported the plausibility of the idea that specific therapeutic advances can offset other medical spending.

This paper addresses these issues by using an improved set of methodologies. The most important methodological change regards the identification of drug innovation. Lichtenberg used the number of years a drug had been on the market to measure drug innovation. This measure is limited, because it implicitly treats all drugs of comparable age as the same, effectively assuming that the pace of innovation is constant across time and therapeutic classes. In contrast, the available evidence indicates that drug innovation often consists of significant breakthroughs where varied firms introduce similar new therapies that replace numerous pre-existing therapies. These new drugs are often comparable to each other even though they may be introduced years apart. Further, these waves do not impact all classes. A wave of major innovation in one class may occur during a period when innovation is relatively slow in another.

Our approach to measuring innovation builds on these facts by examining the cost savings associated with major groups of new drugs. More specifically, between the late 1970s and early to mid-1990s, there were several widely noted breakthroughs in pharmaceutical research that led to substantial new drug classes. These breakthroughs included selective serotonin reuptake inhibitors (SSRIs), statins, angiotensin-converting-enzyme (ACE) inhibitors, histamine type 2 (H2) antagonists, proton pump inhibitors (PPIs), calcium channel blockers (CCBs) and fluoroquinolones. These drug classes are important because each class represents a novel approach to therapy or a unique mode of action. Further, if newer drugs have a significant impact on medical expenditures, as suggested by Lichtenberg[1,2] and challenged by Duggan,[4] then the effects ought to become evident for these important new drug classes. From now on, we will call drugs belonging to breakthrough therapeutic classes "important drugs" and drugs belonging to other therapeutic classes as "other drugs."

These new classes of drugs seemingly focus on new therapeutic methods and provide better treatment. A few examples illustrate this phenomenon. An important milestone for the pharmaceutical industry was the introduction of Tagamet®, which was the first H2 antagonist specifically designed to control acid secretion. These histamine antagonists are prescribed to treat active duodenal ulcers, benign gastric ulcers, gastroesophageal reflux disease, and the prophylaxis of stress-induced ulcers. Other important H2 antagonists include Zantac®, Pepcid® and Axid®, all of which were introduced during the 1980s. These products offered new modes of action, but cost more than previously existing therapies (see below). The issue is, what impact, if any, did the use of these drugs have on overall healthcare expenditures.

This wave of products was followed in the late 1980s by the development of PPIs, which, it is argued, also offered improved treatment. These inhibitors work by suppressing gastric acid secretion through specific inhibition of the hydrogen-potassium ATPase enzyme system at the secretory surface of gastric parietal cells. These drugs are used to treat duodenal and benign gastric ulcers, severe erosive esophagitis and long-term treatment of pathologic hypersecretory conditions. The first PPI, Prilosec®, was introduced in 1989 and achieved substantially significant sales. Other PPIs include Prevacid®, Aciphex® and Protonix®, which were introduced during the second half of 1990s. These drugs also cost more and the issue is whether their use reduces other costs.

There were similar substantial pharmaceutical breakthroughs in several other areas. For instance, SSRIs work as antidepressants by blocking the central nervous system's serotonin uptake. SSRIs offer a novel mode of action and are the treatment of choice for many indications, including depression, panic disorder, obsessive-compulsive disorder, and post-traumatic stress disorders. Their use is aided by their good side-effect profile, efficacy, tolerability, safety in overdose and patient compliance.

Statins similarly provide a novel approach to therapy and mode of action. Statins treat heart disease by lowering cholesterol. More specifically, they control the production of cholesterol by inhibiting an enzyme, HMG-CoA reductase. They also reduce cholesterol by increasing the liver's ability to remove cholesterol from the blood. Statins are especially helpful for patients who have had inadequate response to dietary restrictions of saturated fat and cholesterol.

The other innovations listed above exhibit similar changes in therapeutic method and modes of action. These drugs treat conditions in novel ways, but improved treatment is associated with increased pharmaceutical costs. This paper attempts to determine whether these drugs impact other medical expenditures, and if so, whether there is a net cost saving from their use. We depart methodologically from Lichtenberg[1] by examining whether an entire group of breakthrough drugs reduces costs compared to older, pre-existing therapies.

Our analysis of these drug groupings offers several improvements over prior work. First, we treat all drugs with a similar pharmacology as the same, rather than simply assigning their therapeutic value based on year of introduction. This measure offers substantial improvement over drug age because multiple drugs introduced in a given year need not represent the same level of improvement over existing therapies. Second, our approach recognizes that innovations emerge in waves, and that drugs within a particular group are more similar therapeutically to each other than to other existing drugs or other drugs introduced in the same or different years.

Third, we separately estimate the effects of each of these groups of drugs on drug and non-drug expenditures. Lichtenberg's[1,2] approach implicitly assumes that therapeutic improvements and cost impacts are linear in time and the same across classes. Duggan[4] raises issues about whether Lichtenberg's[1,2] analysis applies generally, or whether there are a few or many areas where newer drugs lead to lower non-drug medical expenditures. Our approach relaxes Lichtenberg's[1,2] assumption and specifically addresses group-by-group whether innovative classes of drugs lower non-drug medical expenditures. This approach will improve the econometrics because it amounts to a more general specification, permitting us to investigate whether all groups affect expenditures in the same way.

Finally, we measure the cost impact of drug use in a more general way than in the existing literature. Using the MEPS data, Lichtenberg[1,2] analyzes the impact of drug use on only contemporaneous medical expenditures, because he investigates costs associated with a single medical event (or record) in the data. This approach is problematic for chronic use drugs because patients (and their doctors) switch drugs regularly, going back and forth between newer and older drugs. Our analysis addresses this issue by separating patients into groups based upon the regularity of their use of particular drugs.

As discussed below, our analysis uses MEPs data, which is organized by medical event. Data for a particular medical event record both drug and non-drug expenditures for that event. While it may be true that usage of a superior drug will reduce contemporaneous medical expenditures, for many drugs, medical cost savings will be distributed over time. For example, cholesterol-reducing drugs (statins) would not likely reduce current expenditures, but instead would lower medical expenditures over time. These characteristics of drug therapy mean that it is important to measure benefits over time. Further, it is important to distinguish between patients who consistently use a breakthrough drug and those who occasionally or randomly use such a drug.

The remainder of the paper is as follows. Section 2 presents the model, and Section 3 presents an overview of the data. Section 4 displays the results and Section 5 concludes.

## 2. Model

The analysis relies on a variant of Lichtenberg's[1-3] models where we use a substantially improved measure of new technologies and tighten the definition of drug use. We measure therapeutic innovation by focusing attention on the major breakthroughs in drug treatment over the last two and a half decades. In particular, we include SSRIs, statins, ACE inhibitors, H2 antagonists, PPIs, CCBs and fluoroquinolones. These drugs are widely used, have larger sales, and are commonly identified as providing substantial therapeutic improvements.

To investigate the cost impact of the use of these drugs, it is important to incorporate the fact that the therapeutic benefit of many drugs will be spread over a period of time. The MEPS data, however, is organized according to individual medical events. Particularly for chronic use drugs, but also for any drug whose benefits will affect future as opposed to contemporaneous health outcomes, it is important to measure cost reductions over time.

To address long-term use, we assigned patients to groups based upon the consistency with which they use drugs from the breakthrough groups, grouping patients into three categories. The first category includes patients who frequently switch back and forth between important drugs and other drugs over time. The second category consists of patients who regularly use new, important drugs. This group includes patients who use the important drugs all the time or those who switch between important drugs and other drugs only once throughout their treatment history, for a particular International Classification of Disease, 9$^{th}$ Revision, Clinical Modification (ICD-9-CM) code. The third category consists of patients who never use important drugs.

Our analysis incorporates each of these methodological improvements in measuring the effects of newer drugs on healthcare outcomes. We also control for the effects of the conditions by incorporating condition duration and the patient's diagnosis, or ICD-9-CM codes.

Our formal model is a variant of Lichtenberg's model:

$$Y^{c}_{ij} = \Phi X_j + \Psi Z_{ij} + \Pi M_i + \xi_{ij}$$

where: $Y^{c}_{ij}$ is the category (c) of either prescription drug expenditure (c=DE) or total non-drug expenditure (c=NDE) associated with the j$^{th}$ prescription consumed by person i. The $X_j$ variables measure important drug usage. $X_1$ takes a value of 1 if the patient regularly used important drugs, and a value of 0 otherwise. $X_2$ takes a value of 1 for patients who never use important drugs, and is 0 otherwise. The omitted category consists of patients who switch multiple times between important drugs and other drugs. $Z_{ij}$ includes dummy variables for conditions described as ICD-9-CM 3-digit diagnosis, for which person i used prescription j, and includes condition durations. $M_i$ includes patient i's income level and demographic variables for sex, race, insurance status, education and age. $\xi_{ij}$ is the disturbance term. $\Phi$, $\Psi$ and $\Pi$ are the set of coefficients to be estimated.

## 3. Data

We use the MEPS data sets from 1996 to 2001. MEPS is co-sponsored by the Agency for Healthcare Research and Quality (AHRQ) and the National Center for Health Statistics. MEPS is a nationally representative survey of healthcare use, expenditures, sources of payments, insurance coverage, and demographic characteristics for the U.S. civilian non-institutionalized population. MEPS consist of three components, including the Household Component (HC), the Medical Provider Component (MPC) and the Insurance Component (IC). These surveys jointly generate exceptionally rich datasets that provide national estimates of the level and distribution of healthcare use and expenditures.

Our data set consists of the first five panels of MEPS from 1996 to 2001. First, we created our data set for each panel separately by merging the HC, MPC and IC files. Then, we calculated the medical expenditures associated with each condition by event type, using the Condition-event Link File. Finally, we used the Medical Care Index (1982 to 1984=0) to express all dollar values in terms of year 2001.

We sorted the MEPS data by patient and condition. Patient records were then sorted into chronological order so that the drug treatment history for each patient/condition could be assessed. Individual patient treatment histories vary substantially. For some patients, physicians frequently change drugs, while for others there are initial changes followed by stability, and for still others there is little change. These differences lead to our three categories of drug use: The control group consists of patients who frequently switch drugs over time; a second category consists of patients who exclusively use important drugs or switch drugs only once; and the third category consists of patients who never use the important drugs.

The next step was to identify conditions where breakthrough drugs were regularly used. This issue is important because the MEPS encounter data appear to include prescriptions that do not match well with the associated ICD-9 (diagnostic) codes. For example, statins will be prescribed "for" ulcers, and H2 antagonists will be prescribed "for" depression. The apparent reason is that patients present with multiple indications (diagnoses) and all codes are not recorded.

This data problem creates issues for cost measurement associated with a medical condition. In particular, when a drug is "used" for an unrelated condition "other medical expenses" will be mismeasured, and these medical expenses cannot be compared to the expenses of conditions for which the drug is normally used. Accordingly, we sought to identify medical conditions for which dugs were regularly used. To address this issue, we restricted our attention to ICD-9 codes where the groups of breakthrough drugs were regularly used. Since the definition of "regularly" is subjective, we carried out our analysis using several alternatives. More specifically, we used all codes where the breakthrough drugs were prescribed for at least 4%, 5% and at least 6% of all encounters. These procedures resulted in separate data sets for each breakthrough drug group. The exception consisted of the H2 antagonists and PPIs. Since these drugs are used to treat largely overlapping groups of conditions, their data sets were combined. Analysis of these various groupings indicates that the central results are not sensitive to the use of the 4%, 5% or 6% cutoff.

Similar to ICD-9 codes, the beginning year of the conditions and the health insurance coverage for each patient are also used. We included four health insurance indicators in our analysis. It is important to note that these health insurance variables are not mutually exclusive. This means that some patients have Medicare and private health insurance, or Medicare and Medicaid. The Medicare variable takes a value of 1, if the patient has Medicare insurance coverage according to MEPs, otherwise it takes value 0. The Medicaid variable takes a value of 1, if the patient has Medicaid insurance coverage, otherwise it takes a value of 0. Similarly, the private insurance variable takes a value of 1, if the patient has private insurance coverage, otherwise it takes a value of 0. A patient was considered to have private health insurance coverage if, at a minimum, that coverage provided benefits for hospital and physician services. The last variable, uninsured, identifies patients who do not have any kind of insurance, taking a value of 1, if the patient is uninsured.

The MEPS data set also provides detailed demographic characteristics of each patient including age, sex, race, educational attainment and income. The age variable represents the age as of the end of the year of interview. MEPS data also includes self-reported race, categorized as one of the following choices: American Indian, Aleut/Eskimo, Asian, Black, White and Other races. The MEPS data sets also provide the total income for each patient.

## 4. Empirical Analysis

The empirical analysis focuses on these different groups of important new drugs, beginning with SSRIs. All of the data presented reflect event level outcomes, meaning average expenditures are per event and the regression coefficients reflect impact per event.

*Selective Serotonin Reuptake Inhibitors*

SSRIs are generally prescribed for the treatment of depressive disorders, obsessive compulsive disorders, social anxiety and panic attack disorders. Depression usually occurs as a result of the lack of stimulation of the recipient neuron at a synapse. To stimulate the recipient cell, SSRIs inhibit the reuptake of serotonin and increase the chance for serotonin to be recognized by the receptors. The increase in serotonin level by the recipient cell is believed to act as a stimulant, counteracting depression and increasing the motivation.

The SSRIs include such well-known drugs as Paxil®, Prozac® and Zoloft®. As noted in Table 1, the first SSRI was fluoxetine, which was introduced in 1987. During the next 15 years, additional SSRIs were introduced including sertraline in 1991, paroxetine in 1992, fluvoxamine in 1994, citalopram in 1998, rabeprazole in 1999 and pantoprazole in 2000.

These drugs created substantial changes in the pharmacological treatment of numerous diseases. SSRIs have been prescribed for a wide range of conditions, including schizophrenic disorders, neurotic disorders, personality disorders and depressive disorders. Table 2 presents summary statistics. These data reveal that SSRIs are prescribed for chronic conditions. The reported treatment length is at least 5 years for 36.8% of the conditions. Healthcare expenditures vary with income, insurance status, sex, race and age. The data indicate that patients' mean age is about 44.5 years and average per capita income is $17,386. SSRIs, when compared to other drugs used for the same conditions, are typically associated with higher drug ($81.40 vs. $53.30), but lower non-drug expenditures ($306.20 vs. $455.40). Table 2 also reveals that on average, SSRIs account for 20.1% of prescriptions in the categories where they are regularly used.

Table 3 presents regression results measuring the impact of SSRIs on drug and non-drug expenditures. Inspection of the results shows that the control variables are generally significant and plausible. From now on, we will present the results for all the samples, but discuss them only for the 5% sample. The results show that income is associated with a small but positive effect on total drug expenditures and has an insignificant negative effect on total non-drug expenditures. Patients enrolled with insurance have higher total drug expenditures than uninsured patients. After separately controlling for breakthrough drugs, patients enrolled in private insurance have significantly higher total non-drug expenditures than all other patients, except those covered by Medicaid.

The empirical results indicate that SSRIs cost more than other drugs, controlling for diagnosis, insurance, and demographics, but reduce non-drug expenditures by substantially more. The omitted drug use category consists of patients who sporadically use breakthrough drugs. Focusing on the 5% sample, the estimated results from Table 3 show that the patients regularly using SSRIs incur higher total drug expenditures of $18.19 compared to the patients sporadically using SSRIs. In contrast, patients who never use SSRIs experience savings in total drug expenditures of $15.08, compared to those who sporadically use SSRIs and save $33.27, compared to those who regularly use SSRIs.

**Table 1. New Drug Classes[1]**

| Molecule Name | Trade Name | App. Year |
|---|---|---|
| ***Selective Serotonin Reuptake Inhibitors*** | | |
| Fluoxetine Hydrochloride | Prozac | 1987 |
| Sertraline Hydrochloride | Zoloft | 1991 |
| Paroxetine Hydrochloride | Paxil | 1992 |
| Fluvoxamine Maleate | Luvox | 1994 |
| Citalopram Hydrobromide | Celexa | 1998 |
| Rabeprazole Sodium | Aciphex | 1999 |
| Pantoprazole | Protonix | 2000 |
| ***Statins*** | | |
| Lovastatin | Mevacor | 1989 |
| Simvastatin | Zocor | 1991 |
| Pravastatin | Pravachol | 1991 |
| Fluvastatin | Lescol | 1993 |
| Atorvastatin | Lipitor | 1996 |
| Cerivastatin Sodium | Baycol | 1997 |
| ***ACE Inhibitors*** | | |
| Captopril | Capoten | 1981 |
| Enalapril Maleate | Vasotec | 1985 |
| Lisinopril | Prinivil | 1987 |
| | Zestril | 1987 |
| Benazepril Hydrochloride | Lotensin | 1991 |
| Fosinopril Sodium | Monopril | 1991 |
| Quinapril Hydrochloride | Accupril | 1991 |
| Ramipril | Altace | 1991 |
| Moexipril Hydrochloride | Univasc | 1995 |
| Trandolapril | Mavik | 1996 |

| Molecule Name | Trade Name | App. Year |
|---|---|---|
| ***H2 Receptor Antagonists*** | | |
| Cimetidine | Tagamet | 1977 |
| Ranitidine | Zantac | 1983 |
| Famotidine | Pepcid | 1986 |
| Nizatidine | Axid | 1988 |
| ***Proton Pump Inhibitors*** | | |
| Omeprazole | Prilosec | 1989 |
| Lansoprazole | Prevacid | 1995 |
| Rabeprazole Sodium | Aciphex | 1999 |
| Pantoprazole | Protonix | 2000 |
| ***Calcium Channel Blockers*** | | |
| Verapamil Hydrochloride | Calan | 1981 |
| | Isoption | 1981 |
| | Verelan | 1981 |
| Nifedipine | Adalat | 1981 |
| | Procardia | 1981 |
| Diltiazem Hydrochloride | Cardizem | 1982 |
| | Tiamate | 1982 |
| | Dilacor | 1982 |
| | Tiazac | 1982 |
| Nicardipine Hydrochloride | Cardene | 1988 |
| Nimodipine | Nimotop | 1988 |
| Isradipine | DynaCirc | 1990 |
| Bepridil Hydrochloride | Vascor | 1990 |
| Felodipine | Plendil | 1991 |
| Amlodipine Maleate | Norvasc | 1992 |
| Nisoldipine | Sular | 1995 |
| ***Fluoroquinolones*** | | |
| Norfloxacin | Noroxin | 1986 |
| Ciprofloxacin | Cipro | 1987 |
| Ofloxacin | Floxin | 1990 |
| Enoxacin | Penetrex | 1991 |
| Lomefloxacin Hydrochloride | Maxaquin | 1992 |
| Sparfloxacin | Zagam | 1996 |
| Levofloxacin | Levaquin | 1996 |
| Gerafloxacin | Raxar | 1997 |
| Travofloxacin Mesylate | Trovan | 1997 |
| Gatifloxacin | Tequin | 1999 |
| Moxifloxacin | Avelox | 1999 |

[1]For more detailed information about each drug class, please check the following sources: 1) Drug Facts and Comparisons 2005, by Facts and Comparisons. 2) Goodman and Gilman's The Pharmacological basis of therapeutics, 10th Edition by McGraw-Hill Companies. 3) Physicians' Desk Reference 2005, by Medical Economics. Note: Trade names are used for identification purposes only and do not imply product endorsement. ACE: angiotensin-converting-enzyme

**Table 2. Summary Statistics for the 5% Sample for Selected Breakthrough Drug Categories**

| | | SSRIs | Statins | ACE Inhibitors | H2 Antagonist PPIs | CCBs | Fluoroquinolones |
|---|---|---|---|---|---|---|---|
| **Year of First Introduction** | | 1987 | 1989 | 1981 | 1977 | 1981 | 1986 |
| **Demographics:** | | | | | | | |
| Mean Age | | 44.5 | 57.6 | 59.5 | 45.2 | 58.6 | 43 |
| Mean Education Years | | 11.3 | 11.5 | 11.1 | 9.8 | 11.1 | 10.5 |
| Mean Income | | $17,386 | $22,883 | $20,218 | $18,468 | $20,752 | $18,525 |
| Percentage of Male Patients | | 32.50% | 47.80% | 43.20% | 41.30% | 43.20% | 28.70% |
| Percentage of White Patients | | 86.90% | 85.40% | 77.70% | 84.20% | 79.40% | 87.60% |
| Percentage of Medicare Enrollees | | 23.90% | 42.10% | 44.90% | 28.10% | 45.40% | 25.60% |
| Percentage of Medicaid Enrollees | | 23.80% | 14.00% | 16.30% | 19.60% | 15.70% | 13.90% |
| Percentage of Private Insurance Enrollees | | 56.00% | 64.50% | 58.00% | 60.20% | 58.90% | 65.10% |
| Percentage of Patients with No Insurance | | 12.20% | 7.60% | 9.20% | 10.80% | 8.90% | 12.10% |
| **Average Healthcare Expenditures ($)** | | | | | | | |
| **Important Drugs:** | Drug Expenditures | $81.40 | $85.10 | $44.50 | $95.20 | $57.70 | $63.20 |
| | Non-drug Expenditures | $306.20 | $714.60 | $833.70 | $696.60 | $662.50 | $832.70 |
| **Other Drugs:** | Drug Expenditures | $53.30 | $42.40 | $43.60 | $41.30 | $41.60 | $29.50 |
| | Non-drug Expenditures | $455.40 | $1,979.40 | $943.80 | $1,688.10 | $1,236.30 | $418.90 |
| **Percentage of Reported Condition Durations** | | | | | | | |
| Less than 1 year | | 17.70% | 40.70% | 19.10% | 37.41% | 22.50% | 57.80% |
| 1 year | | 12.10% | 11.00% | 10.80% | 20.28% | 11.00% | 6.40% |
| 2 years | | 7.20% | 8.90% | 7.40% | 6.71% | 7.00% | 9.40% |
| 3 years | | 7.90% | 6.40% | 6.60% | 4.16% | 6.00% | 3.40% |
| 4 years | | 4.40% | 4.70% | 4.70% | 5.51% | 4.40% | 5.70% |
| 5 years | | 4.60% | 3.40% | 4.80% | 2.62% | 4.60% | 5.50% |
| More than 5 years | | 46.20% | 24.90% | 46.60% | 23.28% | 44.50% | 11.80% |
| **Percentage of Important Drugs** | | 20.10% | 15.40% | 8.40% | 17.70% | 10.10% | 10.00% |
| **Number of Patients in Sample** | | 3,098 | 2,111 | 7,846 | 6,773 | 6,976 | 2,746 |

SSRI: selective serotonin reuptake inhibitors; ACE: angiotensin-converting-enzyme; PPI: proton pump inhibitor; CCB: calcium channel blocker

The results also show that SSRIs significantly decrease total non-drug expenditures. Estimated results from Table 3 show that patients who regularly use SSRIs experience decreased total non-drug expenditures of $402.16, compared to patients who sporadically use SSRIs, controlling therapeutic conditions. These savings are about 20 times the amount of the measured increase in drug expenditures. Patients who regularly use SSRIs also experience considerable savings compared to patients who never use SSRIs. Our results indicate that SSRIs result in large decreases in total health expenditures for the patients who use them.

We also used a t-test to determine whether the coefficients for the impact of important drugs on drug and non-drug expenditures are different from the coefficients on other drugs. This test is used to determine cost savings for those who regularly use important drugs, compared to those who never use them. The statistics in the drug and non-drug expenditure equation are respectively 24.02 and 5.59, indicating that the coefficients on important and other drugs are significantly different from each other in both equations.

**Table 3. Analysis of the Impact of SSRIs on Healthcare Expenditures**

| | 4% Sample | | 5% Sample | | 6% Sample | |
|---|---|---|---|---|---|---|
| **Dependent Variables** | **Total Drug Expenditures** | **Total Non-drug Expenditures** | **Total Drug Expenditures** | **Total Non-drug Expenditures** | **Total Drug Expenditures** | **Total Non-drug Expenditures** |
| **Independent Variables** | | | | | | |
| **Important Drugs** | 18.302 | -370.987 | 18.191 | -402.159 | 18.468 | -399.022 |
| | (1.415)‡ | (51.070)‡ | (1.459)‡ | (55.279)‡ | (1.406)‡ | (57.478)‡ |
| **Other Drugs** | -15.291 | -56.219 | -15.08 | -82.18 | -13.358 | -132.975 |
| | (1.057)‡ | -54.639 | (1.149)‡ | -63.085 | (1.074)‡ | (66.870)† |
| **Income** | 0 | 0 | 0 | -0.001 | 0 | -0.004 |
| | (0.000)* | -0.001 | (0.000)* | -0.001 | (0.000)‡ | (0.001)‡ |
| **Male** | -2.127 | 45.261 | -1.608 | 39.995 | -1.199 | 40.891 |
| | (1.037)† | -49.213 | -1.115 | -55.195 | -1.129 | -60.839 |
| **Private Insurance** | 9.289 | 241.241 | 10.857 | 320.718 | 5.2 | 447.344 |
| | (1.626)‡ | (67.305)‡ | (1.765)‡ | (79.803)‡ | (1.908)‡ | (93.084)‡ |
| **Medicare** | 7.999 | -13.422 | 9.26 | 32.144 | 0.048 | 79.966 |
| | (1.632)‡ | -51.394 | (1.767)‡ | -58.288 | -1.682 | -58.48 |
| **Medicaid** | 9.206 | 14.94 | 11.612 | 66.257 | 5.003 | 145.025 |
| | (1.736)‡ | -53.088 | (1.877)‡ | -61.027 | (1.995)† | (73.700)† |
| **Uninsured** | -5.096 | 94.051 | -6.182 | 51.443 | 0.526 | -109.634 |
| | (2.064)† | -86.773 | (2.244)‡ | -97.827 | -2.314 | -111.148 |
| **Eskimo/Aleut** | -24.23 | -956.797 | -34.307 | -1,116.30 | -33.238 | -1,113.98 |
| | (10.062)† | -592.872 | (10.135)‡ | (657.417)* | (10.101)‡ | (624.080)* |
| **Asian** | -7.696 | -413.666 | -20.515 | -532.736 | -21.094 | -486.633 |
| | -6.428 | -582.748 | (6.482)‡ | -658.77 | (6.219)‡ | -621.746 |
| **Black** | -18.888 | -279.511 | -23.323 | -331.141 | -18.881 | -196.583 |
| | (4.720)‡ | -608.76 | (5.123)‡ | -691.931 | (4.878)‡ | -663.194 |
| **White** | -12.784 | -445.391 | -18.024 | -542.843 | -17.629 | -441.127 |
| | (4.533)‡ | -589.61 | (4.891)‡ | -666.137 | (4.615)‡ | -632.513 |
| **Other Race** | -42.888 | -651.814 | | | | |
| | (6.089)‡ | -595.498 | | | | |
| **Constant** | 47.147 | 160.359 | 40.888 | 163.158 | 53.78 | 287.496 |
| | (15.297)‡ | -977.019 | (16.066)† | -861.747 | (20.145)‡ | -920.635 |
| **Observations** | 28626 | 28626 | 24734 | 24734 | 22034 | 22034 |
| **R-squared** | 0.1 | 0.05 | 0.11 | 0.05 | 0.11 | 0.05 |

Absolute value of robust standard errors in parentheses; *significant at 10%; †significant at 5%; ‡significant at 1%; Note: The estimated coefficients of dummy variables for ICD-9 codes, condition duration, age and education year are suppressed and available upon request;

*Statins*

Statins are prescribed for the treatment of cholesterol reduction. They are especially helpful for patients whose response to dietary restrictions of saturated fat and cholesterol has not been adequate. They control the cholesterol production rate in the body by inhibiting an enzyme, HMG-CoA reductase. They decrease the cholesterol level by slowing down the production of cholesterol and by increasing the liver's ability to remove cholesterol from the blood.

In contrast to the widely used SSRIs, statins are normally prescribed for a relatively narrow range of conditions, including myocardial infarction and other heart disease types. Lovastatin (Mevacor®) was the first statin introduced, receiving marketing approval in 1989. As noted in Table 1, other statins were introduced introduced over time, including Zocor® (1991), Pravachol® (1991) and Lescol® (1993). Table 1 contains a complete list.

Statins are designed to reduce cholesterol. They control the rate of cholesterol production in the body by inhibiting an enzyme, HMG-CoA reductase. They also exert vasculoprotective actions that include improvement of endothelial function, antioxidant properties, stabilization of atherosclerotic plaques, regulation of progenitor cells, inhibition of inflammatory responses and immunomodulatory actions. Stabilization of atherosclerosis will treat and prevent chest pain, heart attack and stroke.

Table 2 presents summary statistics for statins. The data reveals that on average statins account for 15.4% of prescriptions in the categories where they are regularly used. They are generally prescribed for conditions associated with higher cholesterol levels, and they are associated with a moderate treatment period, though this may in part reflect the newness of these drugs. Twenty-nine percent of patients have a treatment period of more than 5 years. Ninety-two percent of patients are insured and 48% of patients are male. Patients' mean age is 58, and average per capita income is $22,883. Statins, when compared to other drugs used for the same conditions, are typically associated with higher drug expenditures ($85.10 vs. $42.40), but lower non-drug expenditures ($714.60 vs. $1,979.40).

Table 4 presents the regression results regarding the impact of statins on drug and non-drug medical expenditures. Focusing attention on the 5% sample, the results for the control variables show that income, insurance status, sex, race and age affect healthcare expenditures. The estimated coefficients of these control variables are generally significant and plausible. These variables moderately affect drug expenditures and have larger effects on non-drug expenditures. Male patients experience increased total non-drug expenditures of $319.30. Privately insured patients experience higher non-drug expenditures, while Medicare and Medicaid enrollees experience lower total non-drug expenditures. Increased income is associated with reduced non-drug expenditures, perhaps because of greater attention to healthcare by the wealthy.

Turning to the impact of the statins on drug expenditures, patients who regularly use them experience increased total drug expenditures of $37.33 over patients sporadically using statins, and an increase of $48.07 in drug expenditures compared to patients who did not use statins. These higher drug expenditures, however, are more than offset by reduced non-drug expenditures. Patients regularly using statins experience reduced non-drug expenditures of $593.90, compared to other patients, noting that there are insignificant differences in this impact between the other two groups of patients. Hence, statins generate non-drug savings on the order of twelve times their incremental costs. This large decrease in non-drug expenditures indicates that statins provide a novel and effective mode of action for the treatment of high-level cholesterol diseases, and save on other medical costs.

As above, we used a t-test to determine whether the coefficients for the impact of important drugs on drug and non-drug expenditures are different from the coefficients on other drugs. The t-test statistics in the drug expenditure equation and non-drug expenditure equation are respectively 21.23 and 4.61, indicating that the coefficients on important drugs and other drugs are significantly different from each other in both equations. These statistics suggest that there is significant cost savings for those who regularly use important drugs as compared to those who never use them.

**Table 4. Analysis of the Impact of Statins on Healthcare Expenditures**

| | | 4% Sample | | 5% Sample | | 6% Sample | |
|---|---|---|---|---|---|---|---|
| Dependent Variables | | Total Drug Expenditures | Total Non-drug Expenditures | Total Drug Expenditures | Total Non-drug Expenditures | Total Drug Expenditures | Total Non-drug Expenditures |
| Independent Variables | | | | | | | |
| Important Drugs | | 33.566 | -675.31 | 37.329 | -593.904 | 33.343 | -425.716 |
| | | (2.299)‡ | (115.157)‡ | (2.371)‡ | (162.861)‡ | (2.440)‡ | (61.138)‡ |
| Other Drugs | | -14.862 | 97.062 | -11.042 | 37.262 | -13.52 | -331.76 |
| | | (1.296)‡ | -103.13 | (1.414)‡ | -146.277 | (1.664)‡ | (65.689)‡ |
| Income | | 0 | -0.014 | 0 | -0.02 | 0 | -0.006 |
| | | (0.000)† | (0.002)‡ | 0 | (0.003)‡ | (0.000)† | (0.001)‡ |
| Male | | 1.98 | -266.732 | 4.681 | 319.302 | 0.302 | 285.951 |
| | | (0.976)† | (95.474)‡ | (1.087)‡ | (121.303)‡ | -1.298 | (62.938)‡ |
| Private Insurance | | 1.384 | 913.68 | -3.903 | 875.618 | -0.608 | 409.109 |
| | | -1.211 | (128.087)‡ | (1.567)† | (188.926)‡ | -1.863 | (120.343)‡ |
| Medicare | | 4.889 | -1,010.38 | -3.442 | -910.944 | 3.608 | -279.853 |
| | | -3.881 | (170.129)‡ | (2.010)* | (210.876)‡ | -2.286 | (81.386)‡ |
| Medicaid | | -1.619 | 458.304 | -3.641 | -246.518 | -3.175 | 490.083 |
| | | -1.229 | (153.790)‡ | (1.777)† | -219.652 | -2.226 | (120.829)‡ |
| Uninsured | | 4.576 | 101.936 | 10.975 | 918.284 | 7.534 | -239.994 |
| | | (2.309)† | -201.666 | (2.520)‡ | (229.114)‡ | (3.024)† | (124.308)* |
| Asian | | 21.485 | 3,581.88 | 24.15 | 3,052.64 | 17.984 | 403.923 |
| | | (4.726)‡ | (980.508)‡ | (6.150)‡ | (618.390)‡ | (7.089)† | (227.139)* |
| Black | | 5.818 | 3,504.10 | 13.476 | 1,914.11 | 6.675 | 528.761 |
| | | (3.361)* | (994.345)‡ | (4.452)‡ | (532.174)‡ | -5.494 | (222.461)† |
| White | | 5.469 | 3,607.75 | 9.092 | 2,208.41 | 3.984 | 254.031 |
| | | (3.270)* | (996.503)‡ | (4.292)† | (516.616)‡ | -5.13 | -211.143 |
| Constant | | 97.006 | 789.981 | 69.863 | -1,398.07 | 60.643 | -213.717 |
| | | (33.015)‡ | -2,723.79 | (36.199)* | -1,580.06 | (36.008)* | -1,441.04 |
| Observations | | 20337 | 20337 | 11589 | 11589 | 7028 | 7028 |
| R-squared | | 0.06 | 0.18 | 0.12 | 0.26 | 0.14 | 0.25 |

Absolute value of robust standard errors in parentheses; *significant at 10%; †significant at 5%; ‡significant at 1%. Note: The estimated coefficients of dummy variables for ICD-9 codes, condition duration, age and education year are suppressed and available upon request.

## 4.3. Ace Inhibitors

ACE inhibitors were first introduced in 1981 when Capoten® (captopril) was marketed. This introduction was followed by the marketing of numerous other ACE inhibitors over the next 15 years, including Vasotec®, Prinivil®, Zestril®, Lotensin®, Accupril® and others.

ACE inhibitors are usually prescribed for the treatment of hypertension and congestive heart failure. These drugs are also used for metabolic diseases and other diseases of the circulatory system. Following a heart attack, the heart muscle may weaken. ACE inhibitors slow the weakening of the heart. They help prevent future heart attacks by blocking an enzyme that causes blood vessels to tighten. As a result, blood pressure decreases and the supply of blood and oxygen to the heart increases. Hypertension, myocardial infarction, heart failure and diabetes mellitus are the most frequently observed conditions where ACE inhibitors are used. They are generally associated with longer treatment periods; 47% of patients have treatment periods of more than 5 years.

Table 2 presents summary statistics. The data reveal that on average ACE inhibitors account for 8.4% of prescriptions in the categories where they are regularly used. ACE inhibitors are generally prescribed for elderly people; the patients' mean age is 59 years, and the average per capita income is approximately $20,218. Forty-three percent of the patients are male and 90% of patients have health insurance. ACE inhibitors, when compared to other drugs used for the same conditions, are associated with slightly lower non-drug expenditures ($833.70 vs. $943.80). The drug expenditures for ACE inhibitors and other drugs are similar ($44.50 vs. $43.60).

Table 5 presents the regression results analyzing the impact of the use of ACE inhibitors on drug and non-drug medical expenditures. The results for the control variables show only a small (if any) impact of income, gender, and insurance coverage on drug expenditures. Race-related variables have a significant impact on both drug and non-drug expenditures, with much larger effects on non-drug expenditures. Gender and insurance coverage have significant effects, statistically and economically, on non-drug medical expenditures in these samples, controlling for drug use.

**Table 5. Analysis of the Impact of ACE Inhibitors on Healthcare Expenditures**

| | 4% Sample | | 5% Sample | | 6% Sample | |
|---|---|---|---|---|---|---|
| Dependent Variables | Total Drug Expenditures | Total Non-drug Expenditures | Total Drug Expenditures | Total Non-drug Expenditures | Total Drug Expenditures | Total Non-drug Expenditures |
| Independent Variables | | | | | | |
| Important Drugs | -2.281 | -507.487 | -1.435 | -592.599 | -1.479 | -626.421 |
| | (1.023)† | (54.630)‡ | -1.277 | (57.402)‡ | -1.277 | (59.679)‡ |
| Other Drugs | 1.536 | -154.202 | 0.707 | -208.558 | 0.819 | -236.283 |
| | (0.455)‡ | (46.208)‡ | -0.464 | (49.691)‡ | (0.490)* | (53.741)‡ |
| Income | 0 | -0.003 | 0 | -0.003 | 0 | -0.003 |
| | (0.000)‡ | (0.001)† | (0.000)‡ | (0.001)† | (0.000)‡ | (0.001)† |
| Male | -0.166 | 175.895 | 0.103 | 207.024 | 0.252 | 245.925 |
| | -0.414 | (55.035)‡ | -0.422 | (60.200)‡ | -0.447 | (64.904)‡ |
| Private Insurance | 4.317 | 437.844 | 3.65 | 426.267 | 3.336 | 403.292 |
| | (0.557)‡ | (53.291)‡ | (0.565)‡ | (56.275)‡ | (0.600)‡ | (61.457)‡ |
| Medicare | 3.322 | 155.355 | 2.977 | 109.198 | 1.893 | 119.93 |
| | (1.176)‡ | (71.415)† | (1.208)† | -75.722 | -1.242 | -79.018 |
| Medicaid | 1.696 | 265.221 | 0.717 | 212.535 | -0.118 | 210.317 |
| | (0.658)‡ | (54.765)‡ | -0.607 | (56.627)‡ | -0.623 | (61.509)‡ |
| Uninsured | 2.268 | -63.769 | 2.779 | -55.191 | 3.459 | -43.463 |
| | (0.936)† | -72.097 | (0.954)‡ | -76.648 | (1.001)‡ | -82.225 |
| Eskimo/Aleut | 27.41 | -509.407 | 31.245 | -300.531 | 32.896 | -209.656 |
| | (9.575)‡ | (187.674)‡ | (9.962)‡ | (180.711)* | (10.001)‡ | -266.979 |
| Asian | 9.332 | 645.375 | 7.934 | 724.499 | 10.115 | 838 |
| | (1.974)‡ | (143.093)‡ | (1.956)‡ | (154.317)‡ | (2.008)‡ | (249.454)‡ |
| Black | 3.608 | 639.396 | 2.805 | 686.494 | 4.776 | 830.818 |
| | (1.620)† | (115.123)‡ | (1.669)* | (121.849)‡ | (1.714)‡ | (234.470)‡ |
| White | 6.821 | 768.987 | 6.437 | 827.827 | 8.75 | 960.7 |
| | (1.567)‡ | (112.243)‡ | (1.630)‡ | (119.982)‡ | (1.666)‡ | (232.861)‡ |
| Other Race | -22.049 | 150.827 | -21.813 | 97.717 | -19.332 | 157.625 |
| | (4.716)‡ | -234.783 | (4.742)‡ | -248.632 | (4.809)‡ | -336.669 |
| Constant | 81.181 | 10,831.98 | 85.689 | 10,971.42 | 99.334 | 8,898.15 |
| | (19.284)‡ | (4,577.772)† | (19.722)‡ | (4,647.565)† | (54.280)* | (4,550.750)* |
| Observations | 84350 | 84350 | 77796 | 77796 | 71101 | 71101 |
| R-squared | 0.02 | 0.09 | 0.02 | 0.09 | 0.02 | 0.09 |

Absolute value of robust standard errors in parentheses; *significant at 10%; †significant at 5%; ‡significant at 1%. Note: The estimated coefficients of dummy variables for ICD-9 codes, condition duration, age and education year are suppressed and available upon request.

ACE inhibitors substantially impact non-drug expenditures, but have little effect on drug expenditures. The coefficients in the drug expenditure equation are small ($1.43) and insignificant. In contrast, patients who regularly use ACE inhibitors experience sharply lower non-drug expenditures. These patients who regularly use ACE inhibitors save approximately $592.60 per encounter, compared to patients who sporadically use ACE inhibitors. Regular users of ACE inhibitors also spend $384.04 less than patients who never use ACE inhibitors (where $384.04=$592.60-208.56). The results indicate that ACE inhibitors are drugs that cost little (if any) more than previously existing therapies, but produce large non-drug cost savings. These newer drugs provide an important contribution to medical cost containment.

We also used a t-test to determine whether the coefficients for the impact of important drugs on drug and non-drug expenditures are different from the coefficients on other drugs. The statistics in the drug and non-drug expenditure equations are, respectively, 1.72 and 7.90, indicating that the coefficients on important drugs and other drugs are significantly different from each other in both equations.

*H2 Receptor Antagonists and Proton Pump Inhibitors*

H2 antagonists are prescribed to treat active duodenal ulcer, benign gastric ulcer, erosive gastroesophageal reflux disease, and prophylaxis of stress induced ulcers. PPIs are usually prescribed for the same treatments offered by H2 antagonists. They work by suppressing the gastric acid secretion through specific inhibition of the hydrogen-potassium ATPase anzyme system at the secretory surface of gastric parietal cells. They are used for short-term treatment of active duodenal ulcer and benign gastric ulcer, severe erosive esophagitis and long-term treatment of pathologic hypersecretory conditions.

We grouped together our analysis of H2 antagonists and PPIs. We use this grouping because the focus of our analysis is to determine the impact of classes of breakthrough drugs on drug and non-drug expenditures, as compared to older therapies. The hypothesis to be investigated in this section is whether the use of H2 antagonists and PPIs has affected medical expenditures, compared to older therapies.

H2 antagonists made their first appearance in 1977 with the introduction of cimetidine (Tagamet®). This introduction was followed by several other H2 antagonists in the mid-1980s, including ranitidine (Zantac®), famotidine (Pepcid®) and mizatidine (Axid®). The first PPI, Prilosec®, was introduced in 1989. Other PPIs were introduced over the following decade, including Prevacid®, Aciphex® and Protonix®.

The H2s and PPIs are prescribed for a broad range of conditions including for example nutritional and metabolic diseases, as well as digestive and genitourinary system diseases. The most frequently observed conditions are diseases of the esophagus, gastric ulcers, stomach function disorders and abdominal hernia.

Summary Statistics are presented in Table 2. These statistics show that H2s and PPIs are generally prescribed for conditions requiring short or medium treatment periods. Only 23% of patients report conditions existing for more than 5 years, while 37% report conditions lasting less than 1 year. Eighty-nine percent of patients are insured and 41% of patients are male. Average age is 45 years and per capita income averages $18,468. H2s and PPIs, when compared to other drugs used for the same conditions, are typically associated with higher drug expenditures ($85.20 vs. $41.30) but significantly lower non-drug expenditures ($696.60 vs. $1,688.10).

Table 6 presents the regression results regarding the impact of the H2s and PPIs on drug and non-drug expenditures. The estimation results show that healthcare expenditures are influenced by the control variables, including income, gender, insurance status, race and education. These variables have significant effects on non-drug expenditures, but modest effects on drug expenditures. Male patients experience higher non-drug expenditures of $1,215.93. Patients with private health insurance incur higher total non-drug expenditures compared to other groups. Increased income decreases total drug and non-drug expenditures. Asian and black patients incurred lower, and white patients incurred higher total non-drug expenditures, compared to American Indian patients.

The results in Table 6 strongly support the proposition that H2s and PPIs are associated with higher drug costs, but substantially lower non-drug medical expenditures. The estimated results from Table 6 show that patients regularly using H2s or PPIs have greater drug expenditures of $40.09, but lower total non-drug expenditures of $1,581 compared to patients sporadically using these products. In contrast, patients who never used H2s or PPIs incurred $61.96 in lower average costs than those regularly using these products (where $61.96=$40.10 – ($21.86)). These patients, however, incurred higher non-drug costs of $430.89. These differences are highly significant. Hence, the results indicate that H2 antagonists and PPIs substantially reduce healthcare costs.

**Table 6. Analysis of the Impact of H2 Antagonists and PPIs on Healthcare Expenditures**

| | 4% Sample | | 5% Sample | | 6% Sample | |
|---|---|---|---|---|---|---|
| **Dependent Variables** | **Total Drug Expenditures** | **Total Non-drug Expenditures** | **Total Drug Expenditures** | **Total Non-drug Expenditures** | **Total Drug Expenditures** | **Total Non-drug Expenditures** |
| **Independent Variables** | | | | | | |
| **Important Drugs** | 40.64 | -1,284.85 | 40.099 | -1,580.87 | 38.696 | -414.079 |
| | (1.745)‡ | (123.597)‡ | (1.894)‡ | (156.447)‡ | (1.869)‡ | (72.331)‡ |
| **Other Drugs** | -16.699 | -817.692 | -21.859 | -1,149.98 | -24.656 | 123.094 |
| | (1.018)‡ | (120.395)‡ | (1.286)‡ | (157.406)‡ | (1.244)‡ | -78.342 |
| **Income** | 0 | -0.013 | 0 | -0.008 | 0 | -0.002 |
| | (0.000)* | (0.002)‡ | (0.000)‡ | (0.002)‡ | 0 | -0.002 |
| **Male** | 2.735 | 935.846 | 2.814 | 1,215.93 | 1.672 | 500.476 |
| | (0.819)‡ | (90.001)‡ | (1.130)† | (108.365)‡ | -1.133 | (66.813)‡ |
| **Private Insurance** | 2.846 | 592.724 | 4.067 | 323.102 | 2.636 | 528.169 |
| | (1.088)‡ | (100.963)‡ | (1.473)‡ | (122.155)‡ | (1.541)* | (118.506)‡ |
| **Medicare** | 0.347 | -548.015 | 1.967 | -203.995 | 3.639 | 30.66 |
| | -1.99 | (118.023)‡ | -3.022 | -140.666 | -3.095 | -109.415 |
| **Medicaid** | 5.51 | -124.458 | 5.623 | -80.889 | 4.412 | 167.632 |
| | (1.303)‡ | -101.326 | (1.695)‡ | -130.81 | (1.562)‡ | -125.468 |
| **Uninsured** | 1.979 | 131.018 | 1.979 | -20.917 | 7.6 | -294.655 |
| | -1.702 | -141.637 | -2.305 | -202.781 | (2.076)‡ | (139.154)† |
| **Eskimo/Aleut** | 0.604 | 1,586.62 | 9.606 | 2,171.63 | 6.696 | 1,617.31 |
| | -5.308 | (654.683)† | -7.874 | (1,281.585)* | -7.675 | -1,323.50 |
| **Asian** | 4.071 | -455.99 | 10.701 | -1,429.76 | 6.946 | -325.968 |
| | -3.452 | -316.553 | (4.410)† | (429.190)‡ | -4.312 | -306.64 |
| **Black** | 2.375 | 354.935 | 6.42 | -217.027 | 6.88 | 195.928 |
| | -2.518 | (212.639)* | (2.941)† | -279.325 | (3.308)† | -300.384 |
| **White** | 5.656 | 705.013 | 9.003 | 375.578 | 6.365 | 85.596 |
| | (2.342)† | (190.673)‡ | (2.650)‡ | -239.838 | (2.964)† | -285.134 |
| **Other Race** | -29.655 | -568.567 | -39.331 | -3,044.62 | -26.067 | 577.065 |
| | (10.013)‡ | -820.18 | (12.634)‡ | (1,377.925)† | (9.876)‡ | -503.403 |
| **Constant** | 25.777 | 29,324.51 | 5.951 | 36,581.19 | 26.474 | 5,861.40 |
| | (7.985)‡ | (4,185.424)‡ | -22.433 | (5,133.497)‡ | -26.02 | (1,832.678)‡ |
| **Observations** | 29899 | 29899 | 19415 | 19415 | 15538 | 15538 |
| **R-squared** | 0.13 | 0.17 | 0.15 | 0.25 | 0.16 | 0.24 |

Absolute value of robust standard errors in parentheses; *significant at 10%; †significant at 5%; ‡significant at 1%; Note: The estimated coefficients of dummy variables for ICD-9 codes, condition duration, age and education year are suppressed and available upon request.

To determine the cost savings for those who regularly use important drugs as compared to those who never use them, we test whether the coefficients for the impact of important drugs on drug and non-drug expenditures are different from the coefficients on other drugs. The t-test statistics in the drug expenditure equation and non-drug expenditure equation are respectively, 36.25 and 4.39, indicating that the coefficients on important drugs and other drugs are significantly different from each other in both equations.

*Calcium Channel-Blockers*

CCBs are antihypertensive and antianginal. They are prescribed for the treatment of angina due to coronary artery spasm, chronic stable angina, hypertension and arrhythmias. They inhibit the movement of calcium ions across the membranes of cardiac and arterial muscle cell to slowdown the velocity of transmission of cardiac impulse, depression of myocardial contractility and dilation of coronary arteries. As a result, they relax blood vessels and increase the supply of blood and oxygen to the heart while reducing its workload. The first CCBs, verapamil (Calan®, Isoption®) and nifedipine (Adalat®, Procardia®) were introduced in 1981. During the next 15 years, additional CCBs were introduced including diltiazem (Cardizem®) in 1982, nicardipine (Cardene®) and nimodipine (Nimotop®) in 1988. Other CCBs are shown in Table 1. CCBs are antihypertensives and are generally prescribed for the treatment of hypertension, chronic stable angina, arrhythmias and heart valve disorders.

The summary statistics in Table 2 show that CCBs are generally prescribed for chronic conditions with a treatment length of at least five years reported by 44% of patients. Ninety percent of patients are insured and 32% are male. Patients' average age is 43 years and per capita income is $20,752. CCBs, when compared at sample means to other drugs used for the same conditions, are typically associated with higher drug expenditures ($57.70 vs. $41.60), but lower non-drug expenditures ($662.50 vs. $1,236.30).

Table 7 presents the regression results measuring the impact of Calcium Channel Blockers on drug and nondrug expenditures. The results for the control variables show that the demographic and insurance variables have some effect on drug expenditures, though these effects are generally small. The control variables generally impact nondrug medical expenditures, and most of these effects are large and significant. Insured patients have significantly higher non-drug expenditures than uninsured patients. Male patients experience higher non-drug expenditures of $135.72, and various population groups experience substantial differences in non-drug expenditures.

The empirical results in Table 7 show that CCBs cost somewhat more than other drugs, but lead to significant reductions in non-drug expenditures. Patients regularly using CCBs experience increased total drug expenditures of $7.92, but decreased total non-drug expenditures of $223.49 compared to patients sporadically using CCBs. Comparing patients regularly using CCBs with patients who never use CCBs, the group never using CCBs on average saves $14.32 (=$(7.92-(-$6.40)) in drug expenditures but experience an increase of $70.89 (=$(223.49-152.60)) in non-drug expenditures. Both the savings in drug costs and the increase in non-drug costs are measured at the medical event level and are highly significant. We also used a t-test to determine whether the coefficients for the impact of important drugs on drug and non-drug expenditures are different from the coefficients on other drugs. The statistics in the drug expenditure equation is highly significant (13.55), indicating that the coefficients on important drugs and other drugs are significantly different from each other. The statistics in the non-drug expenditure equation is not significant (1.09).

### Table 7. Analysis of the Impact of CCBs on Healthcare Expenditures

| | 4% Sample | | 5% Sample | | 6% Sample | |
|---|---|---|---|---|---|---|
| **Dependent Variables** | **Total Drug Expenditures** | **Total Non-drug Expenditures** | **Total Drug Expenditures** | **Total Non-drug Expenditures** | **Total Drug Expenditures** | **Total Non-drug Expenditures** |
| **Independent Variables** | | | | | | |
| **Important Drugs** | 8.648 | -216.385 | 7.92 | -223.494 | 8.305 | -208.329 |
| | (1.093)‡ | (72.518)‡ | (1.112)‡ | (71.687)‡ | (1.104)‡ | (72.084)‡ |
| **Other Drugs** | -6.265 | -102.32 | -6.403 | -152.597 | -5.807 | -148.028 |
| | (0.527)‡ | (55.981)* | (0.560)‡ | (55.764)‡ | (0.529)‡ | (56.325)‡ |
| **Income** | 0 | -0.002 | 0 | -0.001 | 0 | 0 |
| | (0.000)‡ | -0.001 | (0.000)† | -0.001 | (0.000)‡ | -0.001 |
| **Male** | 1.125 | 223.543 | 1.611 | 135.722 | 1.001 | 141.825 |
| | (0.478)† | (61.291)‡ | (0.514)‡ | (66.674)† | (0.500)† | (68.723)† |
| **Private Insurance** | 3.685 | 483.047 | 3.78 | 472.624 | 4.181 | 398.923 |
| | (0.644)‡ | (69.179)‡ | (0.696)‡ | (75.980)‡ | (0.692)‡ | (76.273)‡ |
| **Medicare** | 7.315 | 190.57 | 8.482 | 265.338 | 7.581 | 108.566 |
| | (1.601)‡ | (93.772)† | (1.781)‡ | (103.680)† | (1.715)‡ | -102.307 |
| **Medicaid** | 2.237 | 357.487 | 2.043 | 394.039 | 0.161 | 308.418 |
| | (0.749)‡ | (67.261)‡ | (0.811)† | (72.639)‡ | -0.704 | (72.963)‡ |
| **Uninsured** | -0.167 | -62.885 | 0.7 | -90.948 | 0.101 | -45.111 |
| | -1.171 | -103.623 | -1.167 | -111.173 | -1.178 | -112.45 |
| **Eskimo/Aleut** | 10.948 | -4,242.22 | 12.597 | -4,020.03 | 12.23 | -4,248.22 |
| | -6.837 | (2,573.193)* | (7.631)* | -2,603.13 | -7.697 | -2,596.22 |
| **Asian** | 5.332 | 508.904 | 6.032 | 541.09 | 7.197 | 604.386 |
| | (2.512)† | (162.372)‡ | (2.820)† | (204.674)‡ | (2.898)† | (214.187)‡ |
| **Black** | 3.796 | 816.618 | 5.382 | 792.308 | 3.16 | 677.732 |
| | (2.152)* | (146.488)‡ | (2.509)† | (189.577)‡ | -2.568 | (200.381)‡ |
| **White** | 5.973 | 971.412 | 6.976 | 1,009.73 | 6.06 | 927.35 |
| | (2.131)‡ | (147.666)‡ | (2.486)‡ | (194.320)‡ | (2.568)† | (205.193)‡ |
| **Other Race** | -18.232 | 1,200.09 | -5.667 | 1,551.89 | -6.092 | 1,353.72 |
| | (9.890)* | -1,041.88 | -7.688 | (811.965)* | -9.017 | -841.461 |
| **Constant** | 75.547 | 8,073.43 | 85.452 | 9,913.01 | 95.472 | 9,662.08 |
| | (18.073)‡ | (3,461.455)† | (24.165)‡ | (4,295.244)† | (27.333)‡ | (4,406.881)† |
| | | | | | | |
| **Observations** | 63900 | 63900 | 57695 | 57695 | 55086 | 55086 |
| **R-squared** | 0.03 | 0.12 | 0.03 | 0.13 | 0.03 | 0.13 |

Absolute value of robust standard errors in parentheses; *significant at 10%; †significant at 5%; ‡significant at 1%. Note: The estimated coefficients of dummy variables for ICD-9 Codes, condition duration, age and education year are suppressed and available upon request.

*Fluoroquinolones*

Fluoroquinolones are prescribed for the treatment of infections caused by susceptible gram negative bacteria, such as E. coli. They work by killing bacteria or preventing their growth. The first fluoroquinolone, norfloxacin, was introduced in 1986. During the next decade, other fluoroquinolones were introduced, such as enoxacin in 1991, levofloxacin in 1996 and moxifloxacin in 1999. Table 1 provides the entire list of fluoroquinolones with year of introduction.

Fluoroquinolones are prescribed for a narrow range of conditions, for example, infectious and parasitic diseases of the genitourinary system. Cystitis, a bladder infection, and urinary tract disorders are the two most frequently observed conditions for which fluoroquinolones are used, and they comprise more than half of our observations.

The summary statistics in Table 2 show that fluoroquinolones are generally prescribed for conditions requiring short treatment length and moderate cost. Fifty-seven percent of the reported conditions have a treatment period of less than 1 year. Eighty-eight percent of patients are insured and 29% of the patients are male. Patients' average age is 43, and per capita income is $18,525. Unlike breakthrough drug classes described above, fluoroquinolones are associated with both higher drug expenditures ($63.20 vs. $29.50) and higher non-drug expenditures ($832.70 vs. $418.90).

The regression results for fluoroquinolones are presented in Table 8. These results show that the control variables are generally small in the drug expenditures equation. With the exception of the race variables, the demographic and insurance variables are generally large and significant in the non-drug expenditures equation. Male patients experience increased total non-drug expenditures of $406.39. Income has a small, negative impact on total non-drug expenditures. Uninsured patients face higher total non-drug expenditures of $456.99.

**Table 8. Analysis of the Impact of Fluoroquinolones on Healthcare Expenditures**

| | 4% Sample | | 5% Sample | | 6% Sample | |
|---|---|---|---|---|---|---|
| Dependent Variables | Total Drug Expenditures | Total Non-drug Expenditures | Total Drug Expenditures | Total Non-drug Expenditures | Total Drug Expenditures | Total Non-drug Expenditures |
| Independent Variables | | | | | | |
| Important Drugs | 30.519 | -427.47 | 31.455 | -294.685 | 31.658 | 141.713 |
| | (2.432)‡ | (181.856)† | (2.584)‡ | -190.719 | (2.613)‡ | -182.199 |
| Other Drugs | -9.953 | -539.017 | -9.342 | -436.255 | -11.766 | 7.138 |
| | (1.476)‡ | (126.280)‡ | (1.631)‡ | (132.013)‡ | (1.682)‡ | -85.983 |
| Income | 0 | -0.003 | 0 | -0.004 | 0 | -0.002 |
| | (0.000)† | -0.002 | (0.000)* | (0.001)‡ | 0 | (0.001)* |
| Male | 2.775 | 360.17 | 3.924 | 406.386 | 3.795 | 371.022 |
| | (1.194)† | (85.898)‡ | (1.411)‡ | (98.173)‡ | (1.436)‡ | (99.681)‡ |
| Private Insurance | 1.859 | -87.183 | 3.212 | 23.643 | -1.131 | -125.241 |
| | -1.681 | -90.823 | (1.876)* | -94.109 | -1.71 | -101.563 |
| Medicare | 9.449 | 54.299 | 7.315 | -219.994 | 4.445 | -203.882 |
| | (2.465)‡ | -121.359 | (2.610)‡ | (119.939)* | (2.683)* | (122.716)* |
| Medicaid | 3.6 | -240.935 | 5.792 | -158.528 | 0.36 | -182.731 |
| | (1.967)* | (94.072)† | (2.170)‡ | (89.009)* | -1.925 | (97.901)* |
| Uninsured | -3.096 | 490.554 | -3.916 | 456.987 | -0.299 | 454.147 |
| | -1.975 | (107.074)‡ | (2.193)* | (114.219)‡ | -2.168 | (111.499)‡ |
| Eskimo/Aleut | -3.774 | 71.462 | -2.835 | -369.146 | -4.465 | -290.416 |
| | -5.506 | -247.17 | -5.522 | -328.606 | -5.824 | -237.966 |
| Asian | 13.201 | -79.771 | 14.489 | -313.024 | -3.31 | 528.181 |
| | (5.226)† | -344.039 | (5.570)‡ | -382.705 | -5.359 | -406.173 |
| Black | -1.629 | 339.234 | -2.778 | -32.177 | 0.258 | 182.722 |
| | -3.911 | (182.530)* | -4.105 | -163.494 | -4.088 | -157.907 |
| White | 1.754 | 167.028 | 0.943 | 10.928 | 1.267 | -78.845 |
| | -3.633 | -153.144 | -3.797 | -146.989 | -3.858 | -138.472 |
| Constant | 44.699 | -565.807 | 70.917 | 205.912 | 69.372 | 362.03 |
| | (13.649)‡ | -768.047 | (18.516)‡ | -529.104 | (18.711)‡ | -447.487 |
| Observations | 7337 | 7337 | 6317 | 6317 | 5053 | 5053 |
| R-squared | 0.19 | 0.17 | 0.21 | 0.17 | 0.19 | 0.12 |

Absolute value of robust standard errors in parentheses; *significant at 10%; †significant at 5%; ‡significant at 1%. Note: The estimated coefficients of dummy variables for ICD-9 codes, condition duration, age and education year are suppressed and available upon request.

In contrast to the results for the other breakthrough classes of drugs, the results for the fluoroquinolones do not support the hypothesis of cost savings for nondrug medical expenditures. The results show that fluoroquinolones are more expensive than other drugs - the coefficients indicate that patients who regularly use fluoroquinolones on average pay $31.46 more than patients who sporadically use them and pay $40.90 more than patients who never use them.

There is some weak evidence that there are cost savings from the use of fluoroquinolones compared to some but not all groups. Specifically, the point estimate indicates a cost savings of $295 for patients regularly using fluoroquinolones, as compared to patients who sporadically use them. This difference, however, is not statistically significant. Moreover, non-drug expenditures were lowest for the group that never uses fluoroquinolones.

It is important to note the differences between fluoroquinolones and other drugs under study. Fluoroquinolones are expensive antibiotics, and it may be that for this category expensive, leading edge drugs are used for more severe cases, which may also entail other complementary and expensive treatments. Still, regardless of the reason, the evidence does not point toward non-drug cost savings for this drug class.

We also used a t-test to determine whether the coefficients for the impact of important drugs on drug and non-drug expenditures are different from the coefficients on other drugs. The statistics in the drug expenditure equation is highly significant (16.41) indicating that the coefficients on important drugs and other drugs are significantly different from each other. The statistics in the non-drug expenditure equation is not significant (0.71).

## 5. Conclusion

This paper investigated impact of new drugs and drug classes on overall healthcare expenditures. The paper focused on the level of reductions in total healthcare expenditures drawn from the replacement of other drugs with break-through drug classes. To measure the effect of the important drugs prescribed for a given condition on the expenditures, we developed a model that captures the effect of important drugs on expenditures in a less restrictive way than the extant literature. Our analysis uses a variant of Lichtenberg's[1-3] models where we have substantially improved the measure of new technologies and tightened the definition of drug use.

We measure improvements in drug technology by determining whether drugs belong to selected groups of breakthrough drugs. Our measure captures the fact that innovations emerge in waves and that drugs within a particular group are more similar therapeutically to each other than to other existing drugs, even if the drugs are introduced several years apart. Our analysis also incorporates new definition of drug usage and the measurement period for improved health outcomes to measure benefits over time and to distinguish between patients who consistently use a breakthrough drug as compared to those who sporadically use such drugs, and those who never use them.

We have created three samples for each new drug class. In the first sample, we took the conditions for which, the percentage of important drugs are at least 4% or higher. In the second sample, the percentage of important drugs for a condition is at least 5% or higher and in the third sample, the percentage of important drugs is at least 6%. After creating three samples for each new drug class, we measured the increase in total drug expenditures resulting from replacement of other drugs with drugs belong to breakthrough classes.

drugs belong to breakthrough classes. Our results demonstrate that drugs from breakthrough classes - except ACE inhibitors - are more expensive than other drugs. Overall, the results in this paper support the fact that important drugs are more expensive than other drugs.

Next, we measured the impact of breakthrough drug classes and other drugs on total non-drug medical expenditures. Important drugs significantly decrease total non-drug expenditures for all breakthrough classes, except fluoroquinolones. In general, the reduction in non-drug expenditures is many times larger than the increase in drug expenditures. Hence, these leading drugs, with the exception of fluoroquinolones, substantially reduce overall healthcare expenditures.

## Declaration of Competing Interests

The authors declare that they have no competing interests.
